# Sago‐like appearance of pleura in Tuberculosis

**DOI:** 10.1002/ccr3.2949

**Published:** 2020-05-22

**Authors:** Ashesh Dhungana, Prajowl Shrestha

**Affiliations:** ^1^ Department of Medicine National Academy of Medical Sciences Bir Hospital Kathmandu Nepal

**Keywords:** medical thoracoscopy, pleural effusion, sago‐like nodules, tuberculosis

## Abstract

Medical thoracoscopy is an excellent tool for evaluation of exudative pleural effusion, and sago‐like appearance of parietal pleura is highly specific for tuberculosis.

## Graphical abstract

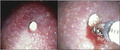

## 1 INTRODUCTION

**Figure 1:**
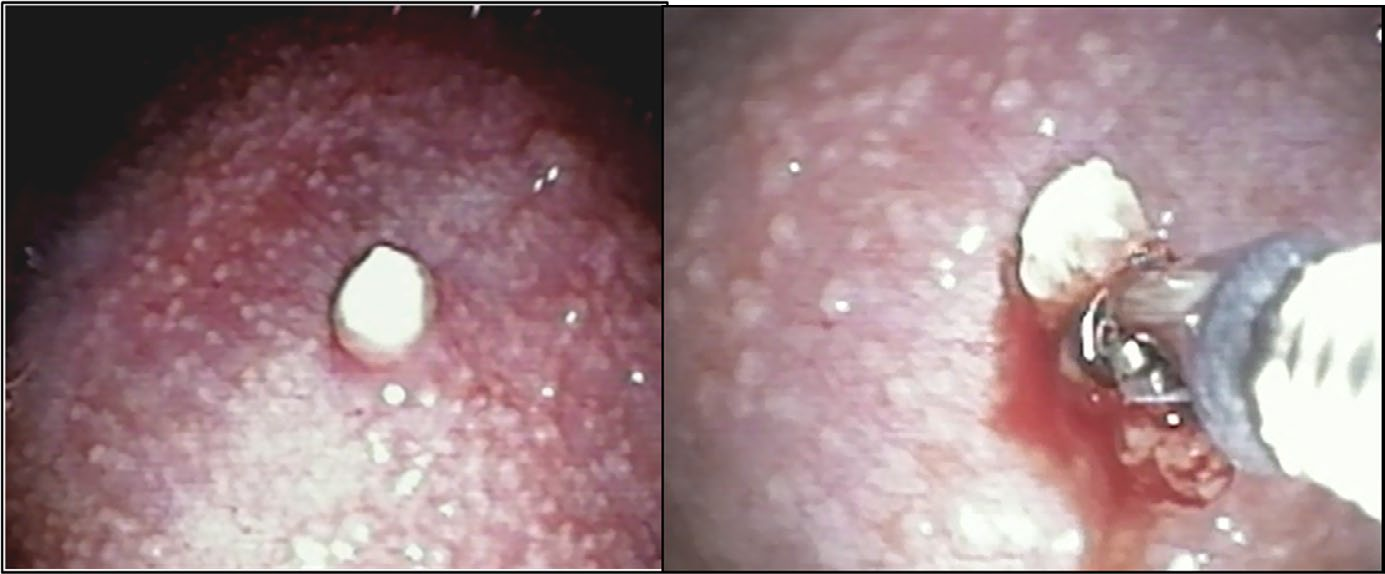
Sago‐like appearance of parietal pleura

A 24‐year‐old lady presented with history of cough, fever, and weight loss for four weeks. Chest radiograph revealed presence of left pleural effusion. Pleural fluid was exudative (protein 3.8 gm %) and lymphocytic (90%) and had low ADA (14 U/L). Cytology revealed no malignant cells. Contrast‐enhanced computed tomography of the thorax showed left‐sided moderate effusion with collapse of left lower lobe. Medical thoracoscopy revealed diffuse thickening of parietal pleura with multiple nodules imparting "sago"‐like appearance (Figure 1). Few nodules had crumbly cheese‐like appearance.

## 2 QUESTION

What is the diagnosis?

## 3 ANSWER

Tuberculosis.

## 4 DISCUSSION

**Figure 2:**
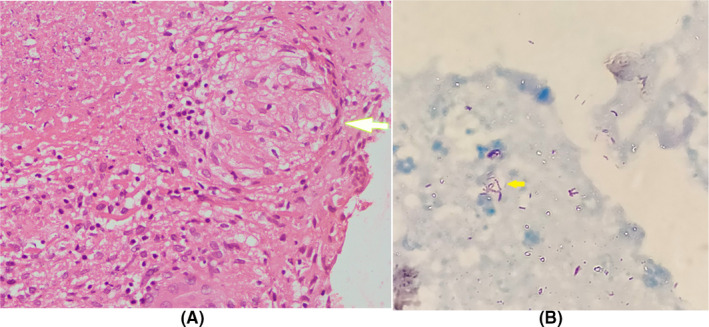
Epitheloid cell granuloma with caseous necrosis (A) and acid fast bacilli (B) in pleural biopsy

“Sago”‐like nodules are the most common visual appearance, and its presence has high specificity and positive predictive value to make a diagnosis of tubercular pleural effusion.^1^ In the index case, thoracoscopic biopsy revealed multiple aggregates of epitheloid cell granulomas with multinucleated giant cells, caseous necrosis (Figure 2A), and multiple acid fast bacilli on Zeihl Neelsen stain (Figure 2B) confirming the diagnosis of tuberculosis. Medical thoracoscopy is an excellent diagnostic modality for exudative pleural effusion, and its yield in tuberculosis is very high.^2^

## CONFLICT OF INTEREST

None of the authors have any conflict of interest.

## AUTHOR CONTRIBUTIONS

AD: involved in concept and design, data acquisition, drafting of manuscript, and critical revision. PS: involved in data acquisition, drafting of manuscript, and critical revision.
